# Isoginkgetin exerts apoptotic effects on A375 melanoma cells

**DOI:** 10.17912/micropub.biology.001324

**Published:** 2024-09-20

**Authors:** Nina Mariano, Hunter Wolf, Pavithra Vivekanand

**Affiliations:** 1 Biology Department, Susquehanna University, Selinsgrove, Pennsylvania, United States

## Abstract

Many plants produce secondary metabolites, known as flavonoids, which are thought to exhibit anti-cancer properties. *Ginkgo biloba* , a plant traditionally used in Chinese herbal medicine, is known to produce over 40 different secondary metabolites. Isoginkgetin, a biflavanoid from this species, has been demonstrated to be cytotoxic to different cancer cell lines. In this study, the anti-cancer effects of isoginkgetin were tested on A375 melanoma cells. XTT cell viability analysis revealed that isoginkgetin treatment resulted in a concentration dependent decrease in cell viability. To investigate whether apoptosis was induced in A375 cell treated with isoginkgetin, a western blot analysis was performed to detect PARP cleavage which is indicative of apoptosis. PARP cleavage was detected at all concentrations tested, with more pronounced cleavage observed with increasing isoginkgetin concentrations. To obtain insight into the potential mechanism of isoginkgetin induced apoptosis, we examined the involvement of the MAPK signaling pathway. We detected phosphorylated ERK in A375 cells treated with isoginkgetin which suggests that isoginkgetin might induce apoptosis of A375 cells through activation of the MAPK signaling pathway.

**Figure 1. Isoginkgetin induces apoptosis of A375 cells:**
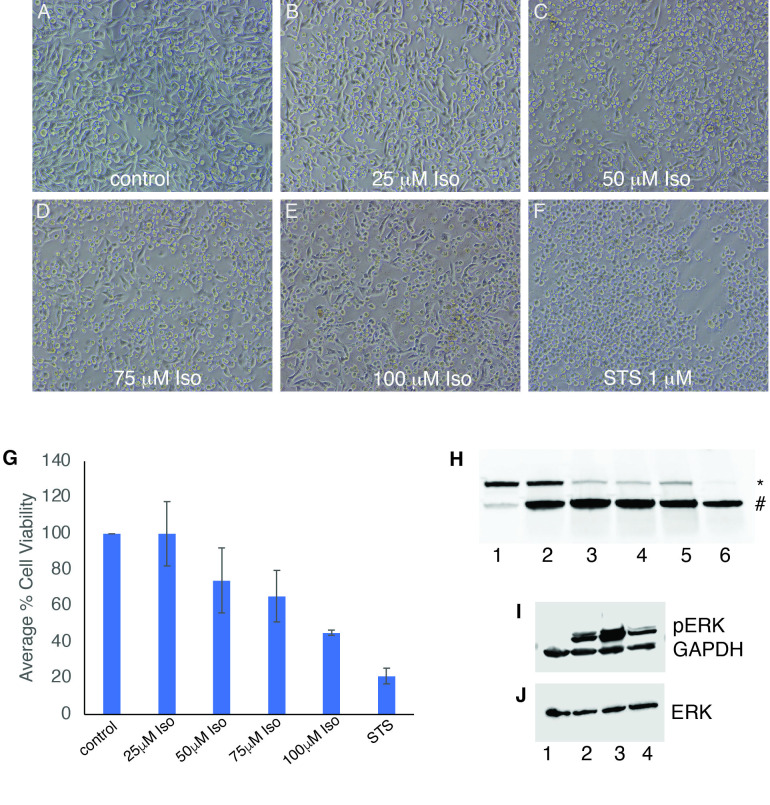
(A) Control cells treated with DMSO exhibit normal morphology, while A375 cells treated with 25-100 μM of isoginkgetin (B-E) or 1 μM staurosporine (F) for 48 hours exhibit apoptotic morphology. XTT cell viability also indicates a concentration dependent decrease in viability in cells treated with increasing concentration of isoginkgetin for 48 hours (G). Data is average of 3 independent experiments +/- SEM. Cells were treated for 24 hours before extraction of protein lysates and western blotting to detect PARP cleavage (H) or phosphorylated ERK and GAPDH (I). PARP cleavage (H) and ERK activation (I) is observed in treated (H, lanes 2-6 and I, lanes 2-4) but not in control cells (H and I lane 1). (J) Total ERK is expressed in both control and treated cells. Western blot results are representative of at least 3 independent experiments. (H) * indicates the 116 kDa full-length PARP, and # indicates the 89 kDa cleaved PARP fragment. (H) Lane 1: control cells (DMSO), Lane 2: 25 μM of isoginkgetin, Lane 3: 50 μM of isoginkgetin, Lane 4: 75 μM of isoginkgetin, Lane 5: 100 μM of isoginkgetin, Lane 6: 1 μM of staurosporine. (I and J) Lane 1: control cells (DMSO), Lane 2: 25 μM of isoginkgetin, Lane 3: 50 μM of isoginkgetin, Lane 4: 1 μM of staurosporine.

## Description

Although cancer is the second leading cause of death globally, given the complexity of the disease, there is a pressing need for the discovery of novel drugs that could be used to treat cancer. While a number of chemotherapeutic drugs have been approved for treatment of malignancies, the effectiveness varies depending on the type of malignancy and underlying genetic causes. Additionally, tumor cells are quick to develop resistance to some chemotherapeutic drugs (Mansoori et al. 2017) . For these reasons, investigating the use of natural sources for novel cancer drugs has been an area of intense investigation, with researchers being particularly interested in the potential anti-cancer properties of plant secondary metabolites such as flavonoids.

The *Ginkgo biloba* plant has been documented to produce over 100 different flavonoids (Liu et al. 2021) . Consumption of flavonoids from the extract of *Ginkgo biloba* (GBE) has shown to exert antioxidant and anti-inflammatory effects which can reduce the risk of cardiovascular disease, cancer, and neurodegenerative diseases (Noor et al. 2022; Biernacka et al. 2023) . 13 unique biflavanoids, which are a subclass of plant flavonoids have been identified in *G.* biloba. Biflavanoids are dimers of flavonoid subunits and have also been reported to have numerous beneficial properties including anti-cancer, anti-bacterial, antioxidant, and anti-inflammatory (Gontijo et al. 2017; Samec et al. 2022) . Of this biflavonoid subclass, isoginkgetin, an isomer of ginkgetin has been demonstrated to exhibit pro-apoptotic and anti-proliferative properties through different mechanisms on a number of human cancer cell lines such as fibrosarcoma, breast carcinoma, and HeLa cervical cells (Yoon et al. 2006; Li et al. 2019; van Zyl et al. 2021) . HeLa cells were most sensitive to the treatment indicating that isoginkgetin is cell specific in potency. Isoginkgetin exhibited a dose dependent increase in cytotoxicity on HeLa cells by upregulating the expression levels of Bax and cleaved caspase-3, both pro-apoptotic regulators (Li et al. 2019) . Further investigation in human fibrosarcoma and breast carcinoma cells showed that isoginkgetin treatment alters tumor invasiveness by inhibiting matrix metalloproteinase 9 (MMP-9) (Yoon et al. 2006) . MMP-9 contributes to cell invasion and is expressed in various malignant tumors. This decrease in MMP-9 levels in cells treated with isoginkgetin was attributed to a concentration dependent downregulation in the activation of the PI3K/AKT signaling pathway (Yoon et al. 2006) . Isoginkgetin has also been shown to act as a pre-mRNA splicing inhibitor (O'Brien et al. 2008) that results in cell cycle arrest at the G1, S, and G2 phases in human colon and ovarian cancer cell lines (Vanzyl et al. 2018) as well as the G2 and M phase arrest in human cervical cancer cells (Li et al. 2019) . Cell cycle arrest of various cancer cells indicates that isogingketin, in addition to inducing apoptosis, inhibits cell proliferation.

Melanoma is the 5 ^th^ most common cancer in the United States (Saginala et al. 2021) . Given the evidence that isoginkgetin exhibits anti-cancer properties, we were interested in examining its effects on a mammalian melanoma (A375) cancer cell line. Although researchers have documented the apoptotic effect of isoginkgetin on many mammalian cancer cell lines, to our knowledge, there is no evidence using a mammalian melanoma cell line which motivated our study using the A375 cell line. Our goal was to compare the cytotoxicity and apoptotic mechanism of isoginkgetin on melanoma cells to those already investigated. We were particularly interested in the role of signaling pathways such as MAPK (Mitogen Activated Protein Kinase) since alterations in this pathway have been demonstrated to induce apoptosis (Cagnol and Chambard 2010; Sugiura et al. 2021) . We determined that isogingketin exhibits anti-cancer effects on A375 cancer cells as well as further establish that this compound holds potential for use as an anti-cancer drug.

To assess whether isoginkgetin can induce morphological changes indicative of apoptosis, A375 cells were treated with increasing concentrations of isoginkgetin. Cells treated with DMSO showed no apparent morphological changes ( Fig. 1A ), while cells treated with Staurosporine (STS) ( Fig. 1F ), a known potent inducer of caspase-mediated apoptosis, exhibit visible morphological changes that were consistent with cells undergoing apoptosis. Similarly, A375 cells treated at 25-100 µM concentrations of isoginkgetin ( Fig. 1B -E) showed apoptotic morphology indicating that cells were triggered to undergo apoptosis. To quantify the effect on cell viability, an XTT cell viability assay was performed on A375 cells that were treated with increasing concentrations of isoginkgetin for 48 hours which resulted in a concentration dependent increase in cytotoxic effect on A375 cells ( Fig. 1G ). Cells treated with 25µM concentration of isoginkgetin averaged the same amount of viability (100%) as the negative control DMSO. Decrease in cell viability of A375 cells was first observed at 50µM concentration with 74% viability which further decreased to 45% in cells that were exposed to 100µM concentration of isoginkgetin.

In order to determine if the decrease in cell viability from isoginkgetin exposure was mediated through caspase activation of apoptosis, we examined the cleavage of PARP. During apoptosis, PARP, a 116-kDa (Fig. IH indicated by *) is cleaved by executioner caspases into an 89-kDa fragment (Fig. IH indicated by #). PARP was found to be expressed as the full length 116-kDa protein in control cells that were treated with DMSO (Fig. IH, lane 1). As expected, in cells treated with STS, PARP was almost completely cleaved into the 89-kDa fragment (Fig.IH, lane 6). In A375 cells exposed to 25µM concentration of isoginkgetin, an increase in the cleaved fragment is evident compared to control cells (lanes 2 vs 1). A further increase in the 89-kDa cleaved PARP fragment with a concomitant decrease in the full-length band was apparent in cells treated with higher isoginkgetin concentrations (lanes 3-5). This indicates that A375 cells treated with isoginkgetin are undergoing apoptosis.

Activation of extracellular signal-regulated kinase (ERK) by phosphorylation downstream of the Receptor Tyrosine Kinase (RTK) signaling pathway has been well documented to result in cell proliferation and survival (Zhang and Liu 2002; Guo et al. 2020). More recently, there have been reports that have demonstrated that ERK activation facilitates apoptosis of cells treated with a number of different drugs (Cagnol and Chambard 2010; Sugiura et al. 2021) . For example, phosphorylated and therefore activated ERK has been shown to lead to cell cycle arrest and apoptosis in fibroblast (NIH3T3), breast cancer (MCF-7), and human keratinocytes (HaCaT) cells treated with etoposide (Tang et al. 2002; Lee et al. 2005) , while cisplatin treatment resulted in ERK activation which was required for apoptosis of HeLa cells (Wang et al. 2000) . Similarly, a number of plant derived molecules have been shown to induce ERK dependent apoptosis (Shih et al. 2002; Nam et al. 2008; Huang et al. 2018) . We therefore decided to examine the phosphorylation status of ERK (phospho-ERK) in cells treated with isoginkgetin for 24 hours compared to control cells. Total ERK was expressed in both control and treated cells ( Fig. 1J ), while phospho-ERK was detected in cells treated with isoginketin (25 or 50 µM) or STS (1 µM) ( Fig. 1I, lanes 2-4) but not in control cells (Fig 1I, lane 1). The timing of ERK activation has been shown to result in either cell survival or apoptosis, with early activation resulting in survival. We did not observe phospho-ERK at 3 or 6 hours after treatment with 50 µM of isoginkgetin, but observed phospho-ERK in cells that were treated with 50 µM isoginkgetin at 12 hours which was enhanced after 24 hours of treatment. Our results thus indicate a possible mechanism of induction of apoptosis by activation of the MAPK pathway in cells treated with isoginkgetin. Whether ERK activation is required for isoginkgetin induced apoptotic cell death of A375 cells requires further research.

## Methods

*Cell Culture*

Melanoma (A375) cancer cells were purchased from ATCC (CRL-1619) and maintained in Dulbecco’s modified eagle medium (DMEM) supplemented with 10% fetal bovine serum (FBS) and penicillin-streptomycin (PS). Cells were maintained in an incubator at 37°C with 5% carbon dioxide.

*XTT Cell Viability Assay*

A375 cells were plated in a 100 µL volume/well in a 96-well plate at a density of 2 x 10 ^5^ cells/mL. Cell density was determined using a hemocytometer. After 24 hours of incubation, cells were treated with either DMSO, isogingketin (25-100 µM), or staurosporine (1µM). Cells were treated in triplicate. The cells were incubated for 48 hours with the drugs, following which the media was aspirated and replaced with 150µL of DMEM+XTT labeling reagent. The cells were incubated with the XTT reagent for 4 hours and the absorbance was measured at 490nm using a microplate reader.

*Protein Extraction and Western Blots*

A375 cells were plated in a 6-well plate at a density of 2 x 10 ^5^ cells/mL in a volume of 2 mL/well. After a 48 hour incubation period, cells were treated with DMSO (control), isogingketin (25-100 µM), or staurosporine (1µM) for 24 hours. After 24 hours, the culture media was aspirated, and protein extraction was performed using 100µL of 1X Laemmli buffer. The samples were sonicated at 30% amplitude for 20 seconds and centrifuged at 4°C and 14,000rpm for 5 minutes. Proteins were separated using a 10% SDS-PAGE and transferred onto a nitrocellulose membrane. The membranes were incubated overnight at 4°C with primary antibodies to detect PARP, phospho-ERK 1/2 (phospho-p44/42 MAPK), ERK 1/2 (p44/42 MAPK), and GAPDH. PARP, p-ERK, and ERK were used at a final concentration of 1:000 while GAPDH was used at 1:5000. The membranes were incubated for 1 hour at room temperature with HRP-conjugated anti-rabbit and anti-mouse antibodies used at 1:5000. phospho-ERK1/2 and GAPDH were simultaneously detected with rabbit anti-ERK and mouse anti-GAPDH antibodies and the membranes were stripped and re-probed with mouse anti-ERK antibody to detect total ERK.

## Reagents

**Table:** 

**Reagent**	**Catalog #**	**Available From**
A375 cells	CRL-1619	ATCC
DMEM	25-501	Genesee Scientific
FBS	B003L53	Thomas Scientific
Penicillin-Streptomycin	15140-122	ThermoFisher
XTT Reagent	4891-025-K	R&D Systems
PARP	9542	Cell Signal
Phospho-p44/42 MAPK	4370	Cell Signal
p44/42 MAPK	9107S	Cell Signal
GAPDH	2G7	DSHB
